# Pharmacoinformatics and Breed-Based De Novo Hybridization Studies to Develop New Neuraminidase Inhibitors as Potential Anti-Influenza Agents

**DOI:** 10.3390/molecules28186678

**Published:** 2023-09-18

**Authors:** Bourougaa Lotfi, Ouassaf Mebarka, Bader Y. Alhatlani, Emad M. Abdallah, Sarkar M. A. Kawsar

**Affiliations:** 1 Group of Computational and Medicinal Chemistry, LMCE Laboratory, University of Biskra, BP 145, Biskra 70700, Algeria; lotfi.bourougaa@univ-biskra.dz; 2 Unit of Scientific Research, Applied College, Qassim University, Buraydah 52571, Saudi Arabia; 3 Department of Science Laboratories, College of Science and Arts, Qassim University, Ar Rass 51921, Saudi Arabia; 140208@qu.edu.sa; 4 Laboratory of Carbohydrate and Nucleoside Chemistry, Department of Chemistry, Faculty of Science, University of Chittagong, Chittagong 4331, Bangladesh; akawsarabe@yahoo.com

**Keywords:** antiviral, flu, neuraminidase inhibitors, molecular docking, pharmacokinetic, molecular dynamics simulation, MM-PBSA, reaction-based enumeration

## Abstract

Influenza represents a profoundly transmissible viral ailment primarily afflicting the respiratory system. Neuraminidase inhibitors constitute a class of antiviral therapeutics employed in the management of influenza. These inhibitors impede the liberation of the viral neuraminidase protein, thereby impeding viral dissemination from the infected cell to host cells. As such, neuraminidase has emerged as a pivotal target for mitigating influenza and its associated complications. Here, we apply a de novo hybridization approach based on a breed-centric methodology to elucidate novel neuraminidase inhibitors. The breed technique amalgamates established ligand frameworks with the shared target, neuraminidase, resulting in innovative inhibitor constructs. Molecular docking analysis revealed that the seven synthesized breed molecules (designated Breeds 1–7) formed more robust complexes with the neuraminidase receptor than conventional clinical neuraminidase inhibitors such as zanamivir, oseltamivir, and peramivir. Pharmacokinetic evaluations of the seven breed molecules (Breeds 1–7) demonstrated favorable bioavailability and optimal permeability, all falling within the specified parameters for human application. Molecular dynamics simulations spanning 100 nanoseconds corroborated the stability of these breed molecules within the active site of neuraminidase, shedding light on their structural dynamics. Binding energy assessments, which were conducted through MM-PBSA analysis, substantiated the enduring complexes formed by the seven types of molecules and the neuraminidase receptor. Last, the investigation employed a reaction-based enumeration technique to ascertain the synthetic pathways for the synthesis of the seven breed molecules.

## 1. Introduction

**Figure 1:**
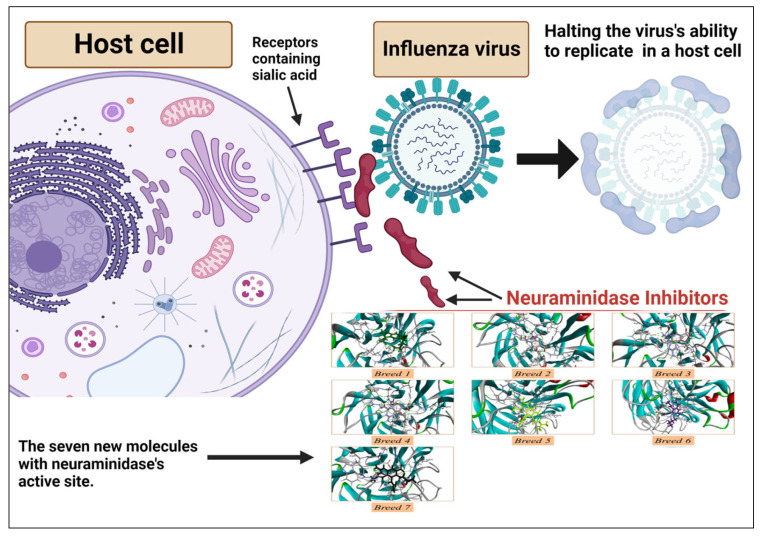
Antiviral mechanism of neuraminidase inhibitors.

Influenza virus infection, commonly known as the flu virus, is an acute respiratory ailment. It frequently manifests in global outbreaks and epidemics, primarily coinciding with the winter season. Substantial quantities of influenza viral particles are discernible within the respiratory excretions of afflicted individuals, thereby facilitating transmission through mechanisms such as sneezing and coughing, which facilitate the dispersion of larger particle-laden droplets [1,2]. Influenza viruses (Orthomyxoviridae family) have segmented negative-sense RNA genomes and are enveloped. Genera A, B, C, and Thogotovirus exist, with A and B relevant to humans. Influenza A/B viruses have eight genome segments that are loosely enclosed by nucleoprotein. Polymerase complexes (PB1, PB2, PA) are at the nucleocapsid ends, surrounded by M1-matrix protein within a host-derived lipid envelope. The glycoproteins hemagglutinin, neuraminidase, and M2-matrix protein are embedded [3]. Annual epidemics of circulating influenza strains cause severe illness and kill between 300,000 and 650,000 people worldwide each year [4]. Managing influenza continues to pose challenges, demanding a comprehensive understanding of available pharmaceuticals and the viability of combination treatments. Effective drug selection hinges on factors including patient age, overall health, and heightened susceptibility to potential complications [5]. Satisfactory management of influenza virus-related issues remains elusive in the absence of an optimal therapeutic intervention. Nonetheless, three registered drug categories explicitly address the influenza virus: M2 proton channel antagonists (amantadine), neuraminidase inhibitors (NAIs; zanamivir, oseltamivir, peramivir, laninamivir), and the recently introduced polymerase acidic endonuclease inhibitor (baloxavir marboxil), which is notable for its novel mechanism of action [6]. Currently, the FDA has approved only two classes of medications for the treatment of different influenza strains and subtypes: matrix-2 (M2) protein ion channel blockers (such as amantadine and rimantadine) and neuraminidase (NA) inhibitors (such as zanamivir and oseltamivir) [7]. Neuraminidase (NA) is a glycoprotein present on the surface of influenza viruses, particularly those of the A and B types. It plays a critical role in the viral life cycle and contributes to the virus’s ability to infect and spread within a host organism. One of the key functions of neuraminidase is related to the virus’s escape from infected cells and its spread to new cells. This is particularly evident during the late stages of viral replication and is related to its enzymatic activity [8] (Figure 1). Moreover, there has also been a marked increase in the emergence of drug-resistant strains in recent years, which has become a major public health issue globally [9,10]. The notable escalation in flu virus resistance to M2 inhibitors in recent years implies that these strains possess a transmission fitness substantial enough to initiate and sustain an epidemic independently. Nonetheless, the scenario is less discernible concerning NA inhibitors [11]. Neuraminidase (NA) is the second major surface protein, playing key roles in the life cycle of influenza virus. NA has an enzymatic function that cleaves terminal sialic acids from glycans, facilitating virus release on the host cell surface [12]. In the 1990s, antiviral agents that directly inhibited NA’s conserved enzymatic active site were developed, and they illustrated efficacy in reducing symptom scores, illness duration, inflammatory markers, and viral titers in human subjects [13]. NAIs are now the most frequently recommended anti-influenza drugs. They have been demonstrated to be effective in accelerating virus clearance, shortening the clinical illness period, and reducing hospital stays and deaths [14,15]. Peramivir, a neuraminidase inhibitor with the same mechanism of action as oseltamivir, has been shown to have activity against both influenza viruses and to shorten the duration of influenza symptoms [16,17]. In this manuscript, we conducted an in silico study using a breed-based de novo hybridization strategy to generate novel neuraminidase inhibitors. Using structural information and the known positions of two ligands, the de novo breed method reconstructs fragments from each ligand to create a new ligand [18]. Breed was recognized to automate this operation via a bond-matching and fragment-swapping system similar to Ho and Marshall [19]. In this manner, it is possible to produce an enormous number of new inhibitors from a small number of starting structures. However, these inhibitors are not simply the side chains of one known inhibitor attached to its scaffold [20]. Additionally, they are not just restricted to combining two distinct scaffolds. Many compounds generated after only two breed crossing iterations, mixing scaffold and side chain components from as many as four of the lead compounds, exhibit no similarity to any of the initial ligand structures [18,21]. The current study aimed to estimate the pharmacokinetic properties and potential toxicity of new breed molecules, and we investigated the stability of the complexes using dynamics simulation and MM-PBSA calculation.

## 2. Results

### 2.1. Breed-Based De Novo and Molecular Docking Approaches

**Figure 2:**
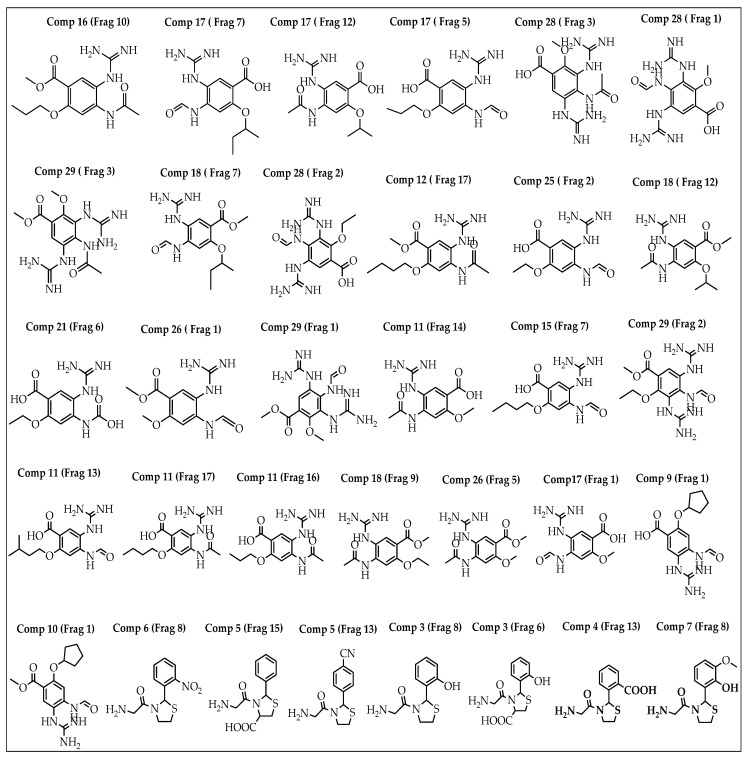
Structures of the best fragments.

**Figure 3:**
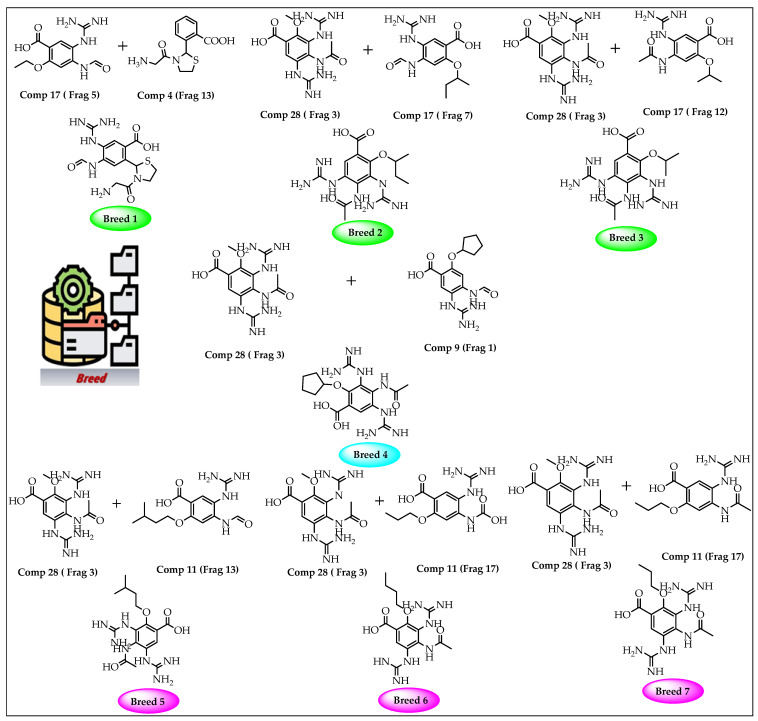
Breed generation of top-scoring compounds from different fragments.

**Figure 4:**
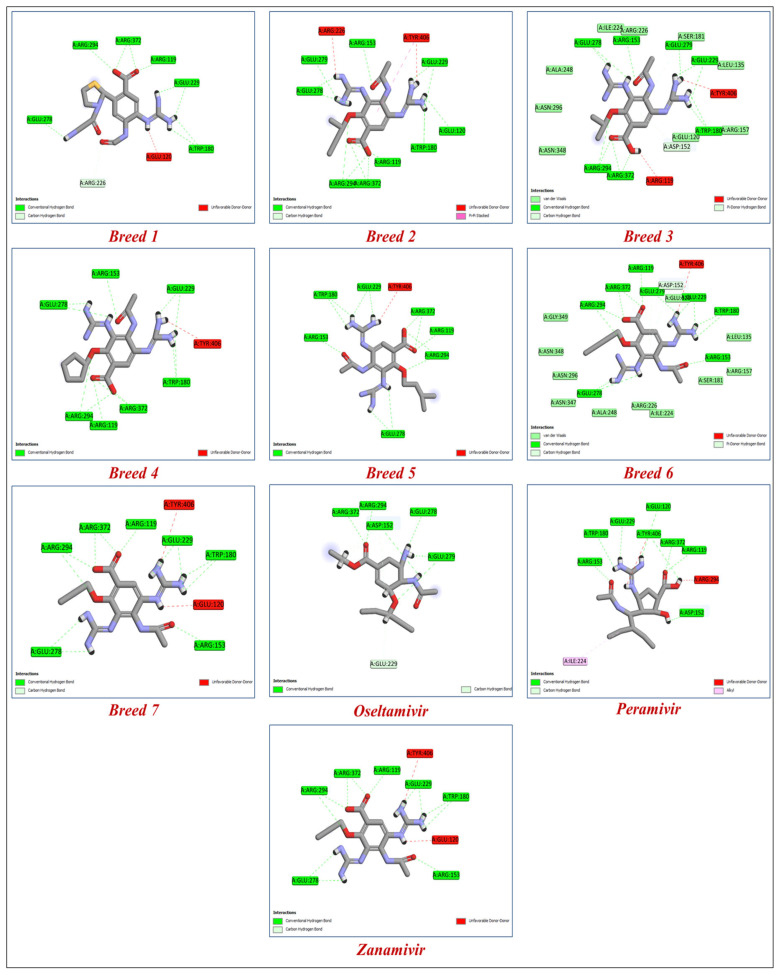
The binding interactions of the seven new breed molecules, zanamivir, peramivir, and oseltamivir within the active site of neuraminidase.

A breed-based de novo strategy was employed in the current investigation to develop new neuraminidase inhibitors. A total of 282 different fragments were generated from thirty potent neuraminidase inhibitors using the Schrödinger PowerShell command “run.\fragment_molecule.py”. We initially docked all the generated fragments (282) in the active site of neuraminidase using SP docking. Figure 2 and Figure 3 show all fragments with docking scores greater than −6 Kcal/mol, while Table 1 reveals the SP docking scores of the top fragments. Compounds 28, 17, 11, 9, 21, 4, 5, 3, and 29 produced high-scoring fragments (between −7.002 and −8.700 Kcal/mol) with the neuraminidase receptor. The breed de novo hybridization approach has now progressed toward fully automating the design process, which could significantly accelerate the procedure and produce better results [20]. The top fragments (with docking scores greater than −6.0) were selected for the breed de novo hybridization process. Subsequently, all 67 novel breed compounds, including zanamivir and clinical neuraminidase inhibitors (oseltamivir and peramivir), were selected for SP rather than the process of XP docking into the active site of neuraminidase. The best breed molecules, with breed scores between 7.886 and 15.623, were as follows: Breed 1 was obtained through hybridization of comp17 (frag5) and comp4 (frag13); Breed 2 was obtained through hybridization of comp28 (frag3) and comp17 (frag7); Breed 3 was obtained through hybridization of comp28 (frag3) and comp17 (frag12); Breed 4 was obtained through hybridization of comp28 (frag3) and comp9 (frag1); Breed 5 was obtained through hybridization of comp28 (frag3) and comp11 (frag13); Breed 6 was obtained through hybridization of comp28 (frag3) and comp11 (frag17); and Breed 7 was obtained through hybridization of comp28 (frag3) and comp11 (frag16), as illustrated in Figure 4.

Antivirals are essential to the control and prevention of influenza. Oseltamivir and zanamivir are the only neuraminidase inhibitor medications that are now licensed globally. Recently, laninamivir and peramivir gained permission in Japan [22]. To know more about how breed molecules inhibit the vital function of the neuraminidase of influenza virus, we docked oseltamivir and peramivir into the neuraminidase receptor. The results obtained indicate that the seven new breed compounds had binding affinity values between −10.529 and −11.867 kcal/mol, and the binding affinity value of reference molecules (zanamivir) was −9.848 kcal/mol, while the clinical inhibitors peramivir and oseltamivir had binding affinity values of −8.844 and −6.326 kcal/mol, respectively. These results demonstrate that the seven breed molecules formed very stable complexes with the active site of neuraminidase. The docking modeling results and the breed score are presented in Table 2. The important information we obtained from this study is that hydrogen-bonding interactions are responsible for inhibitory activity. The amino acids in the active site of neuraminidase that interacted with the seven types of molecules via hydrogen bonds were Arg119, Trp180, and Glu278. The best molecule (Breed 1) interacted with the amino acids Asp152, Arg226, and Glu226 at a distance between 1.61 and 3.33 Å; on the other hand, the reference molecule (zonamivir) interacted with the amino acids Asp152, Glu229, Glu278, Glu279, and Arg372 at a distance between 1.50 and 4.15 Å. This result indicates that the interaction with the amino acids Arg226 and Glu226 plays a very important role in inhibiting the vital function of neuraminidase. Similar to hydrogen bonds formed between molecules (Breed 2–Breed 7) with amino acids Arg119, Trp180, and Glu278, other hydrogen bonds with amino acids Glu120, Arg153, Arg194, Glu229, Glu279, and Arg372, with distances ranging from 1.22 to 5.98 Å, formed stable complexes with the neuraminidase receptor compared to neuraminidase with zanamivir, peramivir, and oseltamivir complexes. Figure 4 shows the binding interactions of the seven tested molecules, zanamivir, peramivir, and oseltamivir, with neuraminidase inhibitors.

### 2.2. ADME-Tox Prediction

The physicochemical characteristics of a compound have a major effect on its pharmacokinetics in the body, and a firm understanding and accurate prediction of these properties are critical for successful drug discovery [23,24]. We estimated the pharmacokinetic properties for the seven breed molecules (Table 3), in particular, the most important parameters, such as LogP, solubility, permeability, and bioavailability, to ensure that the seven breed molecules would reach the biological target (neuraminidase).

The seven breed molecules had molecular masses below 410 g/mol, which facilitated the intestinal absorption of these molecules by oral administration. Second, the partition coefficients (logP) of the breed molecules were between −2.28 and 0.77. These results clearly show the affinity of these molecules toward biological membranes and confirm the good distribution of these substances in the body. For aqueous solubility, all the breed molecules were soluble in aqueous media (including the intracellular medium), and the Log S (ESOL) values were between −0.74 and 0.24. Because of this aqueous solubility, the breed molecules would be easily eliminated through the kidneys. The bioavailability score for Breed 1 was 0.55, while the rest of the breed molecules had the same bioavailability score (0.17). The seven breed molecules did not inhibit the vital function of hepatic enzymes (cytochrome P450), such as CYP 1A2, CYP 2C19, CYP 2C9, CYP 2D6, and CYP 3A4, and they also had Log Kp (skin permeation) values between −8.41 and −11.38. Ultimately, the synthesis of these seven breed molecules in the chemical laboratory is quite simple for in vitro and in vivo research.

The results obtained by ProToxII (Table 4) show that all the breed molecules were safe. The predicted LD50 for the top molecules (Breed 1) was 1098 mg/kg and the toxicity class was 4, while the rest of the breed molecules (Breeds 2–7) had LD50 values between 3200 and 4000 mg/kg and the same toxicity class (class 5). According to the results obtained by the molecular docking and the ADME-Tox study, seven breed molecules (Breeds 1–7) were selected for simulation investigation of their molecular dynamics [23,25].

### 2.3. Study of Molecular Dynamics

Molecular dynamic simulation (MDS) [23] was employed to examine the physical movements of the neuraminidase (NA) and its complexes (Breeds 1–7) during 100 ns of simulations. The root-mean-square deviation (RMSD), root-mean-square fluctuation (RMSF), radius of gyration (Rg), hydrogen bonds (H-bonds), and solvent-accessible surface area (SASA) were calculated to assess the stability of the neuraminidase and its seven complexes (Breeds 1–7).

### 2.4. RMSD and RMSF Analysis

**Figure 5:**
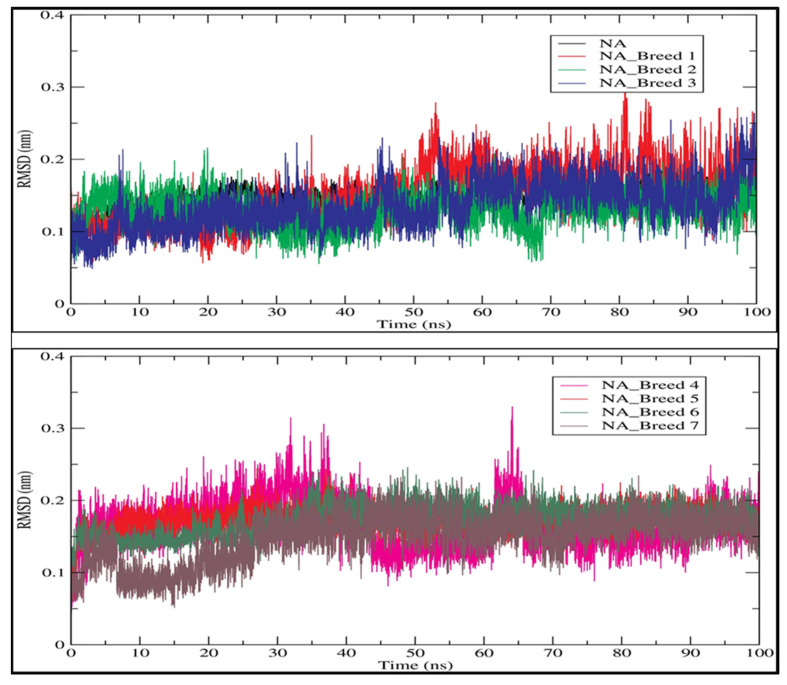
Root-mean-square deviation (RMSD) profile of the backbone atoms of neuraminidase and its complexes with Breeds 1–7.

The RMSD is a factor used to determine the equilibration, protein flexibility, and average distance between backbone atoms of a protein [23]. The RMSD plot for the backbone atoms of the neuraminidase (NA) and its complexes with Breeds 1–7 is displayed in Figure 5. The results indicated that the RMSD of the neuraminidase was stable during 100 ns of simulation, while the NA_Breed 7 complex attained structural stability after 15 ns of simulation.

**Figure 6:**
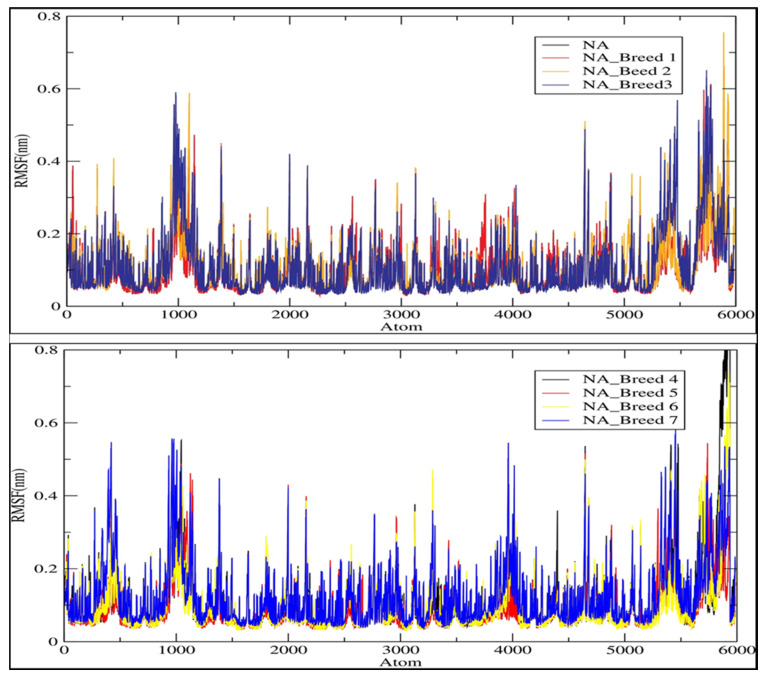
Root-mean-square fluctuation (RMSF) of the C-alpha atoms of neuraminidase (NA) and its complexes with Breeds 1–7.

The rest of the complexes were very stable during the simulation period. The average RMSD values for the NA and its complexes with Breed 1, Breed 2, Breed 3, Breed 4, Breed 5, Breed 6, and Breed 7 were 0.148, 0.149, 0.131, 0.135, 0.166, 0.172, 0.172, and 0.148 nm, respectively. Finally, the RMSD analysis indicated that the MD trajectories were generally stable during the 100 ns of simulation and were able to help in the development of new influenza virus inhibitors. In this research, we calculated the root-mean-square fluctuation (RMSF) of the neuraminidase (NA) and its complexes with Breeds 1–7, as shown in Figure 6. This study showed that more flexibility during the molecular dynamic simulation was shown by higher RMSF values, whereas good stability of the complex was reflected by lower RMSF values. The analysis revealed that the average RMSF for the neuraminidase and its complexes was less than 0.123 nm. The average RMSD for the neuraminidase was 0.106 nm, while neuraminidase complexes (NA Breeds 1–7) had average RMSF values that ranged from 0.100 to 0.123 nm, demonstrating the structural stability and little atomic movement of the neuraminidase and its complexes. On the other hand, the NA Breed 1–7 complexes had higher fluctuations at atoms 450, 1000, 5400, and 5800 of the neuraminidase protein, with RMSF values ranging between 0.4 and 0.6 nm. These findings indicate that the interactions of the breed molecules with the neuraminidase caused conformational changes and increased the protein dynamics in those particular regions. The results obtained (Table 5) indicate that these breed molecules (Breeds 1–7) with the active site of the neuraminidase form good, stable complexes [23].

### 2.5. Radius of Gyration (Rg)

**Figure 7:**
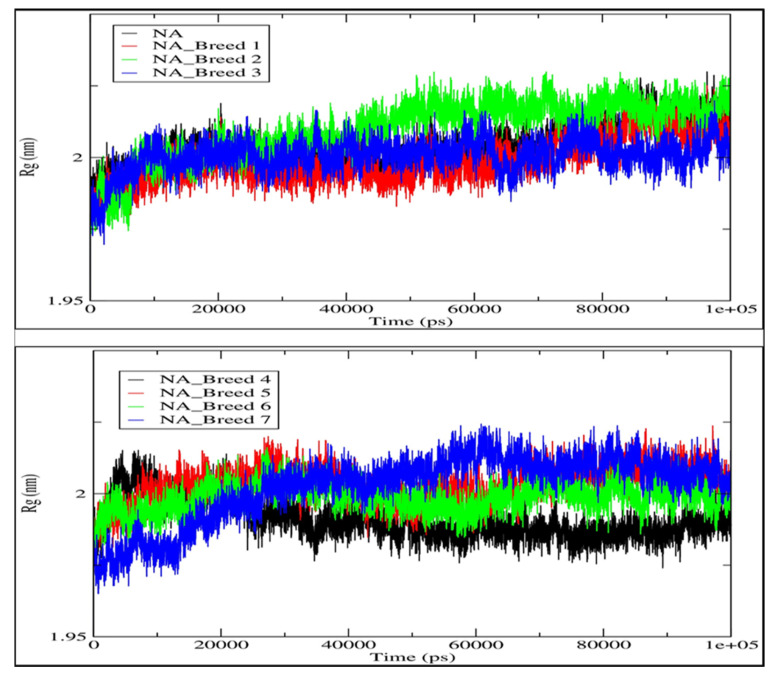
Radii of gyration (Rg) of neuraminidase and its complexes with Breeds 1–7.

We estimated the radius of gyration (Rg) of each system to assess the stability of the neuraminidase and its complexes (NA_Breed 1–7) during the 100 ns simulation (Figure 7). In general, the greater the Rg was, the less compact the Neuraminidase_Breed 1–7 complexes were. During the MD simulation, Rg was employed to determine whether the complexes were stably folded or unfolded. The average Rg value of the neuraminidase was determined to be in the range of 2.003 nm. Moreover, the average Rg values of NA_Breed 1, NA_Breed 2, NA_Breed 3, NA_Breed 4, NA_Breed 5, NA_Breed 6, and NA_Breed 7 complexes were 1.998, 2.009, 2.000, 1.991, 2.003, 1.998, and 2.002 nm, respectively. As previously stated, if a protein maintained a relatively constant value of Rg throughout the MD simulation, it was considered to be stably folded; if its Rg changed over time, it was considered to be unfolded [26,27]. Overall, our results suggest that all the tested molecules formed stable complexes with NA and were able to inhibit the vital function of the neuraminidase.

### 2.6. Hydrogen Bonding Analysis

**Figure 8:**
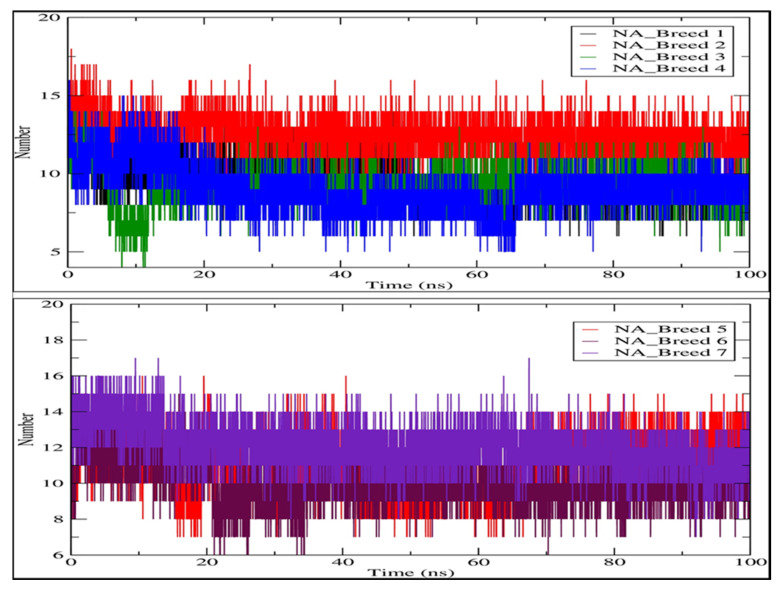
Map of hydrogen bond existence of neuraminidase complexes with Breeds 1–7.

We analyzed the hydrogen bond profiles of the seven complexes to obtain a better understanding of the interaction between the breed molecules and neuraminidase, as shown in Figure 8. The formation of hydrogen bonds between a ligand and a receptor is necessary for the ligand-protein complex to be stable [28]. Our analysis revealed that the NA_Breed 2 and NA_Breed 7 complexes formed averages of 12.444 and 12.064 hydrogen bonds during the simulation. The rest of the complexes formed average hydrogen bonds of between 9 and 11. The existence of more polar groups in the breed molecules (Breed 2 and Breed 7) made it possible to form more hydrogen bonds with the active site of the neuraminidase, in addition to having a strong binding interaction. The rest of the complexes, on the other hand, had some less polar groups and, thus, formed fewer hydrogen bonds with the neuraminidase receptor. This research indicates that hydrogen bonding interaction is essential in the stabilization of the breed molecule with neuraminidase.

### 2.7. Solvent-Accessible Surface Area (SASA)

**Figure 9:**
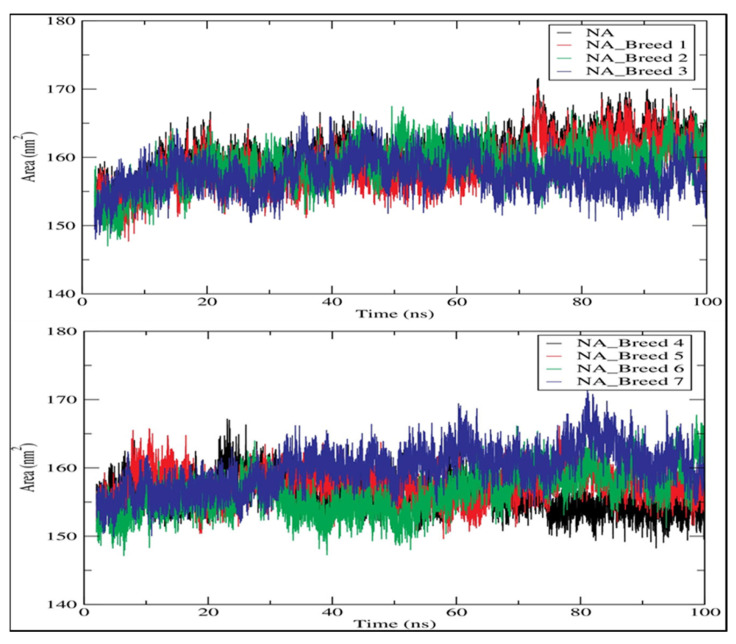
Solvent-accessible surface area (SASA) for neuraminidase and its complexes during 100 ns of simulation.

A greater SASA value shows that the protein volume is expanding, and a low fluctuation is observed over the simulation duration [29]. The average SASA values of all systems were 158.812, 158.835, 157.379, 156.121, 157.397, 156.429, and 159.806 nm2 for NA_Breed 1, NA_Breed 2, NA_Breed 3, NA_Breed 4, NA_Breed 5, NA_Breed 6, and NA_Breed 7, respectively, and 160.245 nm2 for the neuraminidase (Figure 9). All of these findings suggest that the seven complexes were stable over 100 ns of simulation [23].

### 2.8. MM-PBSA Analysis

The binding free energy of the seven complexes (Neuraminidase_Breed 1–7) was estimated using the MM-PBSA approach applied in Gromacs using MD trajectories. The van der Waals interactions (ΔEVDW), electrostatic interactions (ΔEEEL), nonpolar interactions in a solvated system (ΔEPB), nonpolar contribution of repulsive solute–solvent interactions to the solvation energy (ΔENPOLAR), nonpolar contribution of attractive solute–solvent interactions to the solvation energy (ΔEDISPER), total gas-phase molecular mechanics energy (ΔGGAS), and total solvation energy (ΔGSOLV) are all included in the total binding free energy. The total binding energies of the seven complexes were found to be within an acceptable range of between −76.06 and −34.96 KJ/mol. Table 6 shows the MM-PBSA results. With reference to binding affinity with neuraminidase, the NA_Breed 7 complex showed the lowest binding free energy and the highest binding affinity (−76.06 KJ/mol). The binding free energies for NA_Breed 1, NA_Breed 2, NA_Breed 3, NA_Breed 4, NA_Breed 5, and NA_Breed 6 were −55.00, −74.55, −47.61, −44.89, −34.96, and −42.48 KJ/mol, respectively. These free energy calculations confirmed the molecular docking result, demonstrating that these breed molecules interacted with the active site of neuraminidase positively and could be used for the development of new neuraminidase inhibitors.

**Figure 10:**
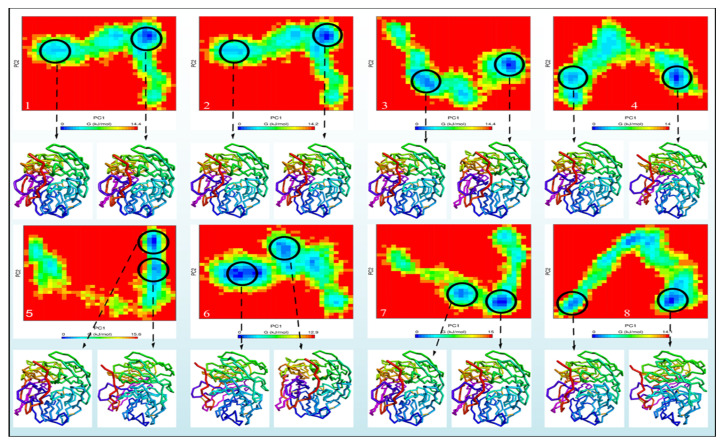

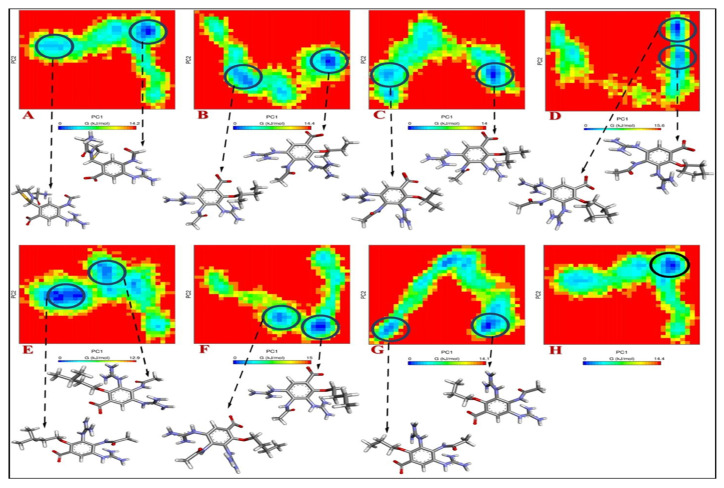
The Gibbs energy landscape maps over 100 ns of simulations for (**A**) NA, (**B**) NA_Breed 1, (**C**) NA_Breed 2, (**D**) NA_Breed 3, (**E**) NA_Breed 4, (**F**) NA_Breed 5, (**G**) NA_Breed 6, and (**H**) NA_Breed 7, with the extracted structures from the low-energy areas (in blue).

Gibbs free-energy landscapes were also produced using the first two PCs to differentiate the conformational modes of the neuraminidase and its complexes (NA Breeds 1–7). The Gibbs free-energy landscapes examined the orientation of the backbone atom fluctuation in neuraminidase and neuraminidase complexes (NA Breeds 7–1) from the MD trajectory. The Gibbs energy landscape plot following 100 ns of simulation, with the extracted structures from low-energy regions for each system, is shown in Figure 10. The results demonstrate that the NA had a Gibbs free energy of 0–14.4 KJ/mol, while the NA_Breed 1–7 complexes had Gibbs free energies of 0–14.2, 0–14.4, 0–14, 0–15.6, 0–12.9, 0–15, and 0–14.1 KJ/mol, respectively.

The blue, cyan, and green areas of the plot represent low-energy states with extremely stable protein conformations, whereas the red area represents a high-energy conformation. The energy landscape had numerous distinct minima that represented metastable structural states separated by a modest energy barrier. The binding of all breed molecules produced the most metastable conformational designs, with regional minima dispersed to approximately two to three areas of the energy landscape. In comparison to neuraminidase alone, the smaller and more concentrated blue minimal-energy regions in NA_Breed 1, NA_Breed 2, and NA_Breed 3 imply extremely stable complexes. Furthermore, the structures (neuraminidase conformations) were found to be similar for all systems. The results demonstrate that the seven types of molecules formed very stable complexes with neuraminidase.

### 2.9. Reaction-Based Enumeration

**Figure 11:**
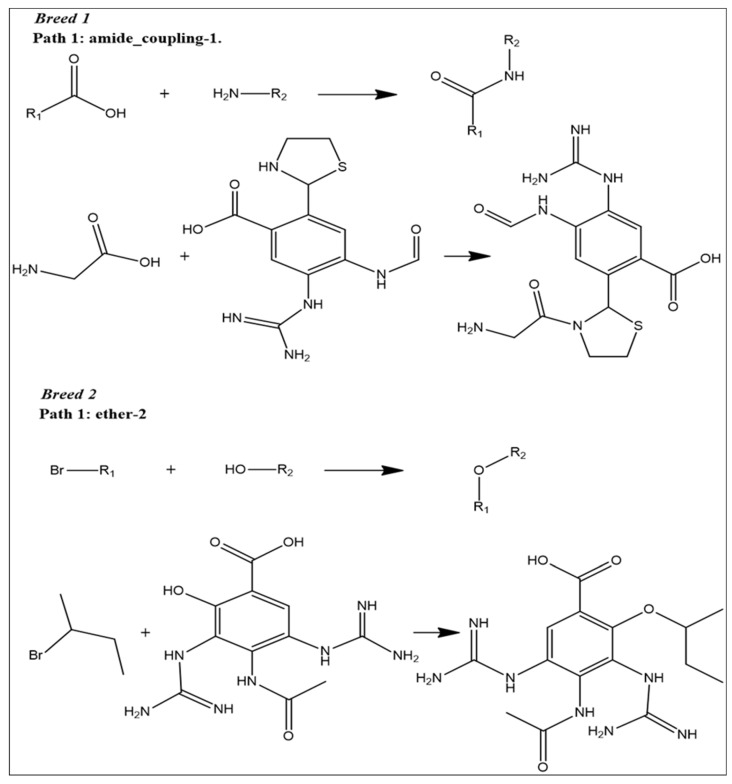

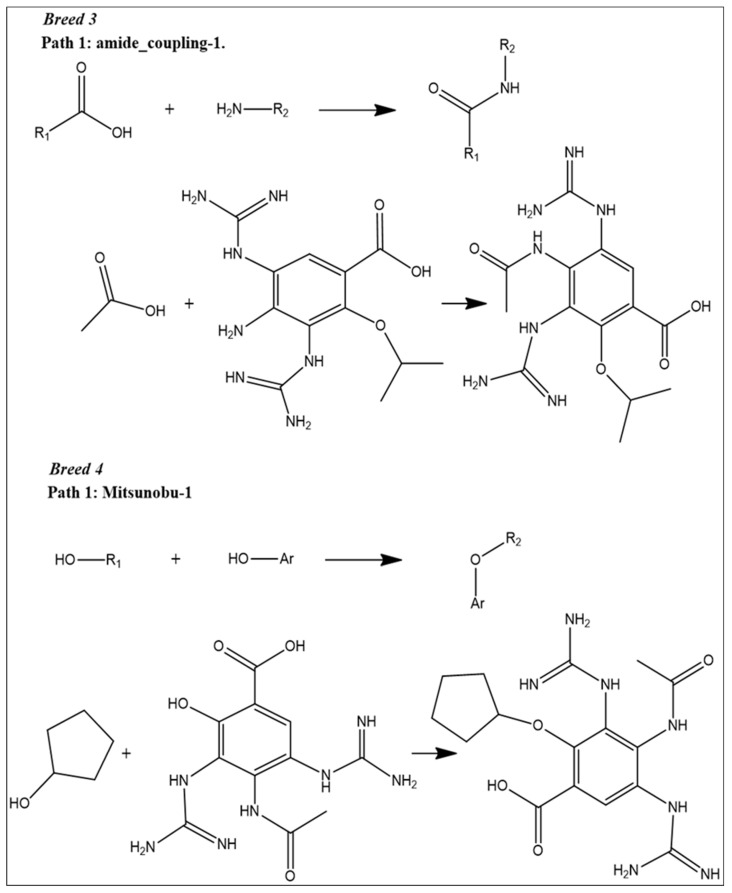

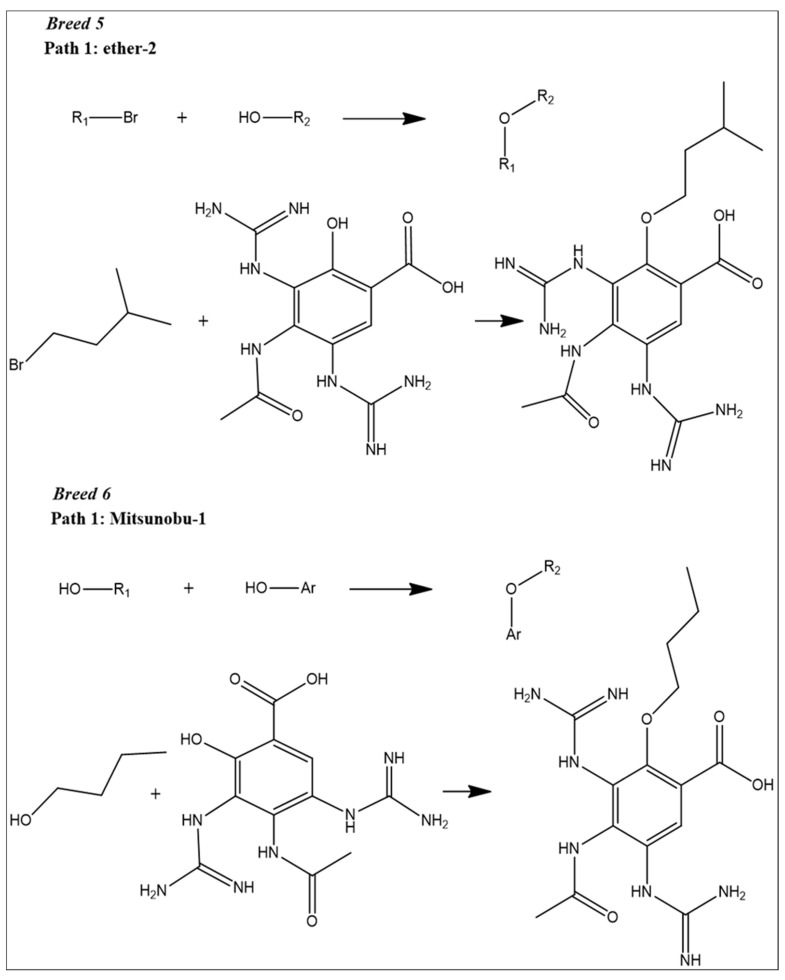

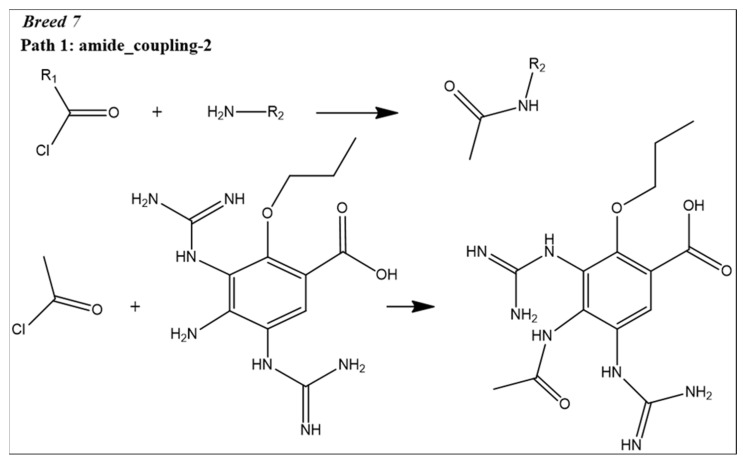
Predicted synthetic pathways of all the breed molecules (Breeds 1–7).

At this step, we used reaction-based enumeration to predict the synthetic pathways of the breed molecules (Breeds 1–7). It is another Schrödinger tool for predicting the synthetic pathway of every molecule using a retro-synthetic methodology. The reaction enumeration tool showed that the amide coupling, ether, and Mitsunobu reactions could be utilized to synthesize all of the breed molecules, as described in Figure 11.

## 3. Discussion

Neuraminidase is a key target in virology and the development of potent anti-influenza medicines. By inhibiting the biological activity of neuraminidase, the influenza virus is prevented from moving to further uninfected host cells and is eliminated. The primary objective was to create prospective novel compounds with anti-influenza activity that is more effective than existing anti-influenza drugs (zanamivir, oseltamivir, and peramivir). Compared with the inhibitory power of clinical neuraminidase inhibitors, all seven designed molecules bound effectively to the neuraminidase receptor, where the proposed molecules attached to other amino acids forming the active site, such as Asp152 and Arg372, which explains the good inhibitory activity of the proposed molecules. The contribution of 2-amino-1-(2-methylthiazolidin-3-yl) ethanone, 2-methoxybutane, 2-methoxypropane, methoxycyclopentane, 1-methoxy-3-methylbutane, 1-methoxybutane, and 1-methoxypropane groups via the interaction of hydrogen bonds can again explain the good inhibitory activity of the developed molecules against neuraminidase. However, these chemical groups had different functions, and they interacted effectively with the essential amino acids that composed the active site, showing that the suggested compounds had a high affinity for the neuraminidase receptor. As pharmacokinetic profiles, the proposed molecules had good permeability toward the membrane bilayer (LogP between −1.55 and 0.24) and good aqueous solubility, which allowed the solubilization of these molecules in the intracellular medium. The hepatic metabolism of the designed compounds was quick and did not produce toxic substances, and no inhibition of cytochrome P450, such as CYP 3A4, was observed. Because of their high water solubility (LogS between −2.28 and 0.77), all of the designed compounds would be quickly removed by the kidneys at the nephron level. In terms of potential toxicity, we found no evidence of any toxicity produced by any of the proposed compounds. All of the aforementioned results indicate that their pharmacokinetic profile is optimal. When we examined the stability of the designed molecules within the neuraminidase receptor using molecular dynamics simulations, we clearly saw insignificant atomic mobility and the continuation of structural stability. During 100 ns of simulation, the seven proposed compounds had average RMSD values ranging from 0.131 to 0.172 nm. In addition, the average RMDF was between 0.1 and 0.123 nm. For the validation of the molecular docking results, the MM-PBSA calculations were in line with the molecular docking study, indicating that all of the molecules proposed formed more stable complexes with the neuraminidase active site, with binding free energies between −76.06 and −34.96 KJ/mol. At the molecular level, Gibbs free-energy landscapes again showed the small dynamic shift of the neuraminidase and its complexes, which confirms the extent of structural stability and the preservation of the initial configuration of all these complexes. Through the experimental examination, our outcomes indicate that the proposed compounds can be synthesized in a chemical laboratory, and it is worth noting that all of the proposed molecules are simple to manufacture using traditional mechanisms. This will make it easier to evaluate and estimate the effectiveness of the designed compounds to inhibit the biological function of neuraminidase in vitro and in vivo. The results of this study will possibly help researchers in the development of anti-influenza medications and virology.

## 4. Materials and Methods

### 4.1. Breed De Novo Hybridization Approach

**Figure 12:**
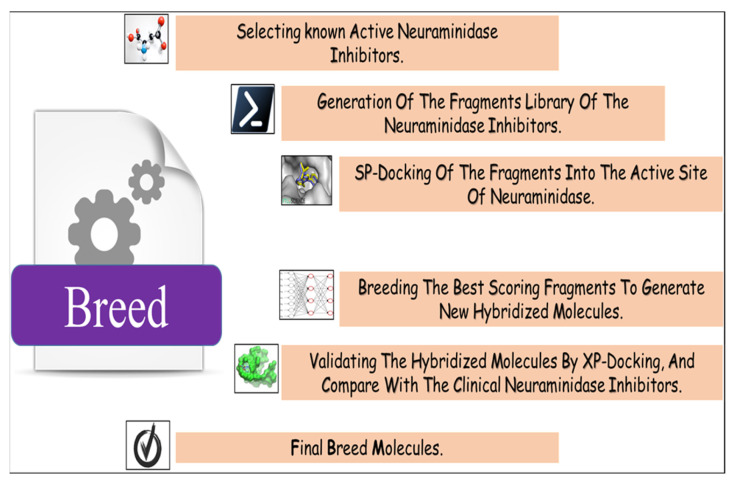
Breed-based de novo hybridization strategy.

**Figure 13:**
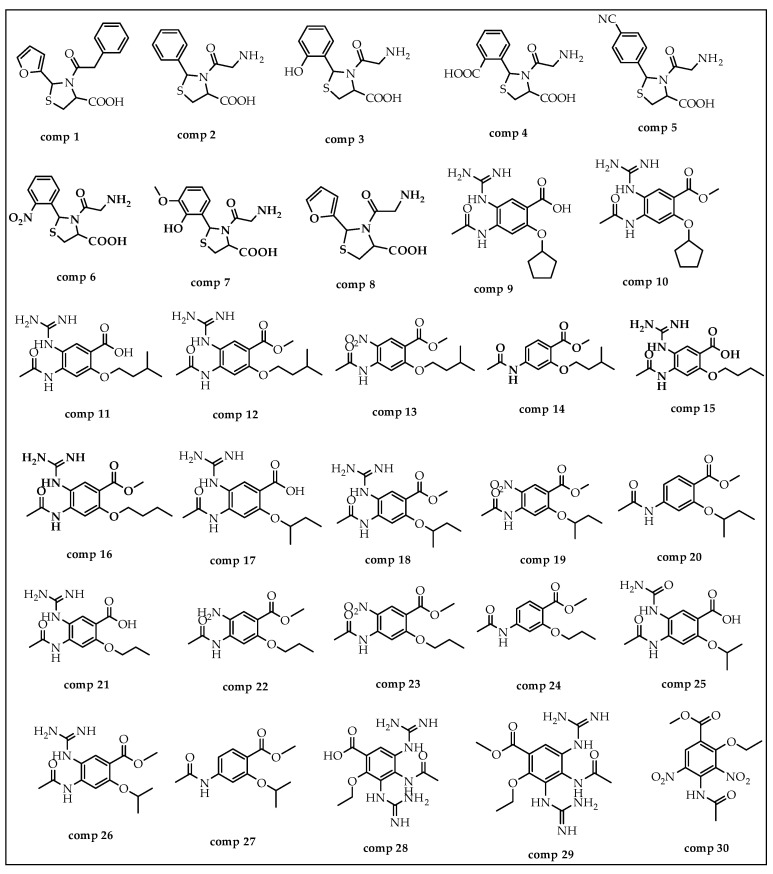
The structures of compounds utilized in this research.

In this step, we used Maestro software (Maestro, version 11.8, 2018, Schrödinger, New York, NY, USA). In breed-based de novo drug design, cocrystalized compounds from diverse PDB structures or well-known inhibitors against a particular target must be used [18]. Numerous neuraminidase inhibitors from the literature were gathered for this investigation (Figure 12) [30,31]. The LigPrep module, which created the low-energy ligand conformer using the OPLS3e force field [32], optimized the 3D structures of 30 compounds. Diverse fragments were produced from these structures using the run.\fragment_molecule.py program at the Schrödinger powershell command line with the appropriate input and output folders. The fragments were docked into the active site of the receptor using SP docking, and the best-scoring fragments were combined using the Breed Ligand Creation Panel [18]. The molecule was hybridized by a breed panel, utilizing three different settings. It should be recalled that two bonds must be of the same order to start and maintain the hybridization (geometry) of the bonded atoms in the new molecule. Additionally, the atoms at the bond ends need to be close to one Å of another [18]. According to Pierce et al. [18], the angle between the bond vectors of two bonds cannot be greater than 15°. The first half of molecule one and the second half of molecule two combine to form a new molecule if the initial molecules are divided in half at the matching bond. The other new molecule is created by joining the first half of molecule two and the second half of molecule one. The atom types, sites, and bonds of the new molecules are identical to those of the corresponding atoms [19]. The procedure for drug discovery, in general, is a difficult challenge for organic chemists due to the complexity of the pharmacophore feature that increases the properties and efficiency of a drug [33]. Chemists commonly use their specialized knowledge and carry out compound tweaking by hand by adding and removing functional groups. Nevertheless, chemists must carry out each alteration step by hand even if they use methods that forecast the ideal chemical attributes. Every iteration of this process might take many hours, and there might still be no promising candidate medication [18]. The breed-based de novo hybridization approach used in this investigation is shown in Figure 13.

### 4.2. Molecular Docking Study

The crystal structure of neuraminidase with zanamivir was downloaded from the Protein Data Bank (PDB ID: 5L17) with a resolution of 2.40 Å and zero mutations [34]. The “protein preparation wizard” in Maestro 11.8 was used to prepare proteins. Hydrogen was added following verification of the chemical accuracy.

The process of energy minimization was used to create a protein with a lower energy state with the help of the OPLS3e force field (optimized potential for liquid simulation) [22]. The grid was constructed using the default box dimension in the SP-docking study. For extensive analysis and validation of the de novo drug design results, XP docking was employed to study the interactions of the new breed of molecules and the active site of neuraminidase.

Finally, we docked two clinical neuraminidase inhibitors (peramivir and oseltamivir) as supplementary reference ligands in the active site of neuraminidase to estimate the inhibitory activity of the breed molecules.

### 4.3. ADME-Tox Prediction

ADMET properties are a very helpful drug discovery approach [35]. The experimental determination of drug candidate pharmacokinetic properties is time-consuming. We investigated the ADMET properties of each breed of molecule, focusing on solubility and permeability to biological membranes, which are two of the most important factors influencing the activity using SwissADME [36]. The ProTox-II platform was used to evaluate the potential toxicity [37]. To study hepatotoxicity, immunotoxicity, and cytotoxicity, the lethal dosage (LD_50_) was determined for both active and inactive cell types [38].

### 4.4. Molecular Dynamic Simulation

In this phase, we conducted molecular dynamic (MD) simulations for all breed molecules. The first process consisted of identifying compounds with high binding affinities for neuraminidase and good ADMET properties. The Gromacs-2022.4 package and the CHARMM27 force field were used to execute the production MD simulation for 100 ns [39]. The SwissParam server was employed for generation of the ligand topology files [40]. Each system was then solvated with the TIP3P water model, and Na+ and Cl− ions were introduced to neutralize the charge [39]. Using the steepest descent-minimization technique, the solvated system was then utilized to minimize energy until the maximal force was less than 10.0 kJ/mol. The equilibration process was then divided into two phases: NVT and NPT equilibrations. The system was coupled with a v-rescale algorithm at 300 K for 100 ps with a coupling value of 0.1 ps during the NVT equilibration phase. The NPT was then equilibrated for 100 ps using a Berenson pressure-coupling strategy with a coupling constant of 2.0 ps [41].

The commands gmx rms, gmx rmsf, gmx gyrate, gmx hbond, and gmx sasa were used to determine the various parameters for each system. The root-mean-square deviation (RMSD) was used to quantify the structural stability of the protein-ligand complex, and the flexibility of the protein residues was assessed using the root-mean-square fluctuation (RMSF) [42]. During 100 ns of simulation, the radius of gyration (Rg) was calculated to assess the compactness of the protein-ligand complex, hydrogen bond analysis was performed to determine the protein-ligand hydrogen bonding interactions, and the solvent-accessible surface (SASA) area was computed to assess the overall stability of each system. In addition, we conducted free-energy landscape analysis, which involves calculating and diagonalizing the covariance matrix [43]. Grace software was employed to visualize the simulation trajectories [44].

### 4.5. Binding Free Energy

For the molecular mechanics Poisson–Boltzmann surface area (MM-PBSA), the protocol implemented in the g_mmpbsa package was used to perform the analysis of the binding free energy (ΔG bind) [45]. The MM-PBSA calculation provides a quantitative prediction of the interactions between proteins and ligand compounds. The binding free energy was calculated as follows:

ΔG bind = G complex − (G protein + G ligand)(1)

where ΔG bind is the total binding energy of the complex, G complex is the binding energy of the native protein, and G complex is the binding energy of the ligand.

### 4.6. Reaction-Based Enumeration

Reaction-based enumeration is another Schrödinger tool that predicts the synthetic pathway of any compound using a retro-synthetic approach [46]. The current study used reaction-based enumeration to establish the synthetic route of the final breed compounds.

## 5. Conclusions

Developing new neuraminidase inhibitors is vital for combating evolving flu strains, enhancing treatment options, reducing resistance, and safeguarding global public health against influenza outbreaks. The objective of this research was to identify novel inhibitor molecules against the neuraminidase of influenza. To develop novel neuraminidase inhibitors, we used a breed-based de novo approach. According to the docking studies, seven breed molecules (Breeds 1–7) demonstrated high stability within the neuraminidase receptor compared to the clinical neuraminidase inhibitors (zonamivir, oseltamivir, and peramivir). On the other hand, to reach the pharmacological target, the pharmacokinetics of the seven breed compounds were investigated, and the results show that they have excellent pharmacokinetic profiles, such as bioavailability and permeability toward biological membranes. The molecular dynamics simulations for 100 ns revealed that the seven breed molecules (Breeds 1–7) were particularly stable in the active site of neuraminidase. In addition, MM-PBSA computations demonstrated that the complexes were very stable throughout the duration of the simulation. This research can contribute to the development of novel and potent neuraminidase inhibitor drugs for the treatment of influenza and could give researchers the opportunity to examine these breed molecules for the treatment of influenza and its symptoms. Finally, the future of neuraminidase inhibitors for the flu involves improved efficacy, reduced resistance, and personalized treatments, aiding in better management and prevention of influenza outbreaks.

## Figures and Tables

**Table 1:** SP docking results for the best fragments.

Fragment	SP-Score (kcal/mol)	Fragment	SP-Score (kcal/mol)	Fragment	SP-Score (kcal/mol)
Comp28 (Frag3)	−8.700	Comp5 (Frag15)	−7.085	Comp26 (Frag5)	−6.425
Comp28 (Frag1)	−8.425	Comp17 (Frag1)	−7.055	Comp6 (Frag8)	−6.370
Comp17 (Frag12)	−8.030	Comp17 (Frag7)	−7.048	Comp18 (Frag9)	−6.264
Comp11 (Frag16)	−7.897	Comp3 (Frag8)	−7.030	Comp16 (Frag10)	−6.117
Comp9 (Frag1)	−7.679	Comp29 (Frag1)	−7.002	Comp18 (Frag12)	−6.166
Comp17 (Frag5)	−7.661	Comp25 (Frag2)	−6.960	Comp12 (Frag17)	−6.144
Comp28 (Frag2)	−7.654	Comp29 (Frag3)	−6.883	Comp26 (Frag1)	−6.135
Comp11 (Frag14)	−7.409	Comp11 (Frag13)	−6.883	Comp5 (Frag13)	−6.073
Comp11 (Frag17)	−7.296	Comp29 (Frag2)	−6.743	Comp15 (Frag7)	−6.042
Comp21 (Frag6)	−7.287	Comp7 (Frag8)	−6.518	Comp10 (Frag1)	−6.031
Comp4 (Frag13)	−7.263	Comp18 (Frag7)	−6.464	Comp3 (Frag6)	−6.012

**Table 2:** Docking results of the breed molecules and zanamivir, peramivir, and oseltamivir with the neuraminidase receptor.

Molecules	Breed Score	SP-Score	XP-Score	H-Bond Interactions	Distance
Breed 1	15.623	−8.799	−11.867	Arg119, Asp152, Trp180, Arg226, Glu226, Glu278, Arg294, Arg372.	1.61–3.33
Breed 2	11.080	−8.372	−10.804	Arg119, Glu120, Arg153, Trp180, Glu229, Glu278, Glu279, Arg294, Arg372.	1.57–5.98
Breed 3	9.952	−8.316	−10.791	Arg119, Asp152, Arg153, Trp180, Glu229, Glu278, Glu279, Arg294, Arg372.	1.48–3.99
Breed 4	13.604	−8.278	−10.765	Arg119, Asp152, Arg153, Trp180, Glu229, Glu278, Glu279, Arg294, Arg372.	1.50–4.91
Breed 5	7.886	−8.457	−10.706	Arg119, Asp152, Arg153, Trp180, Arg194, Glu229, Glu278, Glu279, Arg372.	1.22–3.81
Breed 6	8.298	−7.949	−10.628	Arg119, Asp152, Arg153, Trp180, Glu229, Glu278, Glu279, Arg294, Arg372.	1.48–3.99
Breed 7	10.740	−7.890	−10.529	Arg119, Arg152, Arg153, Trp180, Glu229, Glu278, Arg294, Arg372.	1.57–4.15
*Zanamivir*	-	−7.610	−9.848	Arg119, Arg152, Arg153, Trp180, Glu229, Glu278, Glu279, Arg294, Arg372.	1.50–4.15
*Peramivir*	-	−7.370	−8.844	Arg119, Glu120, Asp152, Arg153, Trp180, Glu229, Glu279, Arg294, Arg372.	1.55–4.39
*Oseltamivir*	-	−5.588	−6.326	Asp152, Glu229, Glu278, Glu279, Arg294, Arg372.	1.50–4.15

**Table 3:** Pharmacokinetic and physicochemical parameters for the seven breed molecules.

Molecules	MW (g/mol)	Log S (ESOL)	Consensus Log P	Cytochrome P450 Inhibitors	Bioavailability Score	Log Kp cm/s	Synthetic Accessibility
Breed 1	366.40	0.77	−1.55	No	0.55	−11.38	3.60
Breed 2	365.39	−1.18	−0.40	No	0.17	−9.02	3.79
Breed 3	351.36	−0.83	−0.74	No	0.17	−9.31	3.19
Breed 4	409.48	−2.28	0.24	No	0.17	−8.41	3.78
Breed 5	379.41	−1.42	−0.13	No	0.17	−8.85	3.46
Breed 6	365.39	−1.07	−0.43	No	0.17	−9.08	3.35
Breed 7	351.36	−0.83	−0.59	No	0.17	−9.24	3.24

**Table 4:** Toxicity prediction of breed molecules.

Molecules	Cytotoxicity	Carcinogenicity	Mutagenicity	Immunotoxicity	Toxicity Class	LD50 (mg/kg)
Breed 1	Inactive	Inactive	Inactive	Inactive	4	1098
Breed 2	Inactive	Inactive	Inactive	Inactive	5	3200
Breed 3	Inactive	Inactive	Inactive	Inactive	5	3200
Breed 4	Inactive	Inactive	Inactive	Inactive	5	4000
Breed 5	Inactive	Inactive	Inactive	Inactive	5	3200
Breed 6	Inactive	Inactive	Inactive	Inactive	5	3200
Breed 7	Inactive	Inactive	Inactive	Inactive	5	3200

**Table 5:** The average values of various parameters, including RMSD, RMSF, Rg, and H-bonds.

Complex	Average RMSD (nm)	Average RMSF (nm)	Average Rg (nm)	Average H-Bonds (nm)	SASA (nm^2^)
NA_Breed 1	0.149	0.105	1.998	9.428	158.812
NA_Breed 2	0.131	0.109	2.009	12.444	158.835
NA_Breed 3	0.135	0.110	2.000	9.196	157.379
NA_Breed 4	0.166	0.115	1.991	9.018	156.121
NA_Breed 5	0.172	0.100	2.003	11.057	157.397
NA_Breed 6	0.172	0.106	1.998	9.964	156.429
NA_Breed 7	0.148	0.123	2.002	12.064	159.806
NA	0.148	0.106	2.003	-	160.245

**Table 6:** Table representing the ΔE_VDW_, ΔE_EEL_, ΔE_PB_, ΔE_NPOLAR_, ΔE_DISPER_**,** ΔG_GAS,_ ΔG_SOLV_, and binding energy for Neuraminidase_Breed 1–7 complexes.

Protein-Ligand Complexes	ΔE_VDW_ (KJ/mol)	ΔE_EEL_ (KJ/mol)	ΔE_PB_ (KJ/mol)	ΔE_NPOLAR_ (KJ/mol)	ΔE_DISPER_ (KJ/mol)	ΔG_GAS_ (KJ/mol)	ΔG_SOLV_ (KJ/mol)	Δ_TOTAL_ (KJ/mol)
NA_Breed 1	−11.61	−385.46	319.53	−25.45	47.99	−397.07	342.07	−55.00
NA_Breed 2	−15.34	−400.88	320.91	−29.39	50.15	−416.22	341.67	−74.55
NA_Breed 3	−12.39	−363.90	306.55	−27.48	49.61	−376.28	328.67	−47.61
NA_Breed 4	−17.29	−347.68	297.99	−29.23	51.32	−364.97	320.08	−44.89
NA_Breed 5	−19.90	−328.15	290.05	−29.25	52.29	−348.05	313.10	−34.96
NA_Breed 6	−24.00	−338.25	297.31	−28.89	51.35	−362.25	319.77	−42.48
NA_Breed 7	−10.87	−407.23	321.76	−28.41	48.69	−418.10	342.04	−76.06

## Data Availability

The data from this study are available from the corresponding authors upon reasonable request.
